# Strong Tree-Size but Weak Shade–Temperature Associations: Fruit-Ball Load and Paired Thermal Contrasts in Shanghai *Platanus* × *acerifolia* Street Trees

**DOI:** 10.3390/plants15213380

**Published:** 2026-11-01

**Authors:** Jiyou Zhu, Hongyuan Li

**Affiliations:** College of Environmental Science and Engineering, Nankai University, No. 38 Tongyan Road, Jinnan District, Tianjin 300350, China; eialee@nankai.edu.cn

**Keywords:** *Platanus* × *acerifolia*, fruit-ball count, fruit hair, tree diameter, negative-binomial regression, paired temperature difference, urban forestry

## Abstract

We examined recorded reproductive traits and paired temperature differences for 200 *Platanus* × *acerifolia* street trees at 50 Shanghai sites. The site-level urbanization index defined 17 low-, 16 medium- and 17 high-urbanization sites. Fruit-ball load was analysed as a whole-number count using negative-binomial regression with site-clustered inference. A one-standard-deviation increase in diameter at breast height was associated with a higher fruit-ball count (incidence-rate ratio 1.419, 95% CI 1.385–1.454), whereas the site urbanization index (*p* = 0.228) and pruning-event frequency (*p* = 0.576) were not independently associated with counts. In 200 paired temperature records, the open-minus-under-canopy difference averaged 5.49 ± 3.73 °C; a 0–8 °C sensitivity subset contained 143 observations. The shade-cover–temperature correlation was −0.070 in the full sample and −0.021 in the restricted subset, providing no evidence of a consistent positive relationship. A descriptive four-quadrant display combines directly counted fruit balls with the restricted temperature differences. Calculated fruit-hair mass is distinguished from measured airborne concentration or human exposure. These results inform interpretation of tree-level reproductive load while placing explicit limits on the use of single-occasion thermal observations for management.

## 1. Introduction

Urban trees contribute to heat adaptation, human well-being and the design of usable streetscapes. Tree crowns intercept radiation and modify near-surface temperatures; the size of these benefits, however, depends on local microclimate, surface cover and the precise measurement being used [1,2,3,4,5,6]. Urban tree management therefore requires more than estimates of green cover alone. Seasonal litter and reproductive structures also affect cleaning demands and residents’ experience [7,8,9,10]. Shanghai’s London plane trees (*Platanus* × *acerifolia*) illustrate the tension between maintaining extensive shade and managing the release of fruit-associated hairs.

Recent work has emphasized variation in canopy-related cooling across streets, surfaces and neighbourhoods [3,4,5,6,11,12,13,14,15]. The 3-30-300 benchmark is useful for urban greening targets, but its individual components may not covary [11]. Equally, an instantaneous difference between a shaded point and an open point is not equivalent to a daily cooling service or a measure of pedestrian thermal comfort. That distinction is essential when field measurements are made without sufficiently documented timing, radiation and reference conditions.

Ecosystem disservices are not simply the inverse of ecosystem services. Cleaning costs, biological debris, allergenic materials and pruning logistics may arise through different pathways from those responsible for shade [7,8,9,10]. A candidate tree should therefore be described along multiple dimensions rather than placed on an assumed service–disservice trade-off. An operational index would ideally distinguish material produced by the tree from the movement of that material through the street and its ultimate human or maintenance consequences.

Seasonal release of fruit hairs from *Platanus* fruit balls is a recognized management concern in Shanghai. Municipal and district communications describe selective removal of fruit-bearing branches as well as physical blowing, washing and road sweeping [16,17,18]. Pruning-event frequency, however, does not reveal whether fruit-bearing branches were removed or how much reproductive tissue was affected. Published pollen and allergen studies [19,20,21,22,23] inform the broader bioaerosol context, but pollen and achene-associated hairs are distinct materials [24]. Here, “fruit hair” refers specifically to fibres associated with the achenes forming a *Platanus* fruit ball; “trichome” is retained only where it corresponds to a recorded microscopic trait.

Studies of urban cooling commonly examine crowns, shading and thermal conditions, whereas studies of nuisance frequently consider reproductive material or exposure separately. A tree-level fruit-ball inventory provides a simple descriptor of potential reproductive material, but cannot quantify airborne fibres or resident exposure. We therefore analyse directly recorded fruit-ball counts independently of derived mass indicators, and use urbanization descriptors as site context rather than a surrogate for tree production.

This study addresses two questions: (1) whether DBH is associated with fruit-ball count after accounting for site-level pruning frequency and urbanization context; and (2) whether the recorded shade-cover percentage is associated with paired open-minus-under-canopy temperature differences consistently across three analytical treatments. A supplementary sample-median display describes the joint distribution of fruit-ball count and temperature contrast. The observational design does not estimate the causal effect of pruning or the health consequences of fruit-hair release.

## 2. Materials and Methods

### 2.1. Study Design, Temporal Scope and Data Provenance

The observational inventory comprised 200 *Platanus* × *acerifolia* street trees nested within 50 Shanghai sites (four trees per site). The study records identify July 2025 as the principal inventory period. Sampling was stratified across low-, medium- and high-urbanization contexts. According to the field eligibility description, four apparently healthy trees were selected at each site, excluding trees with severe visible pest or disease symptoms and using a minimum DBH of 10 cm. Previous heavy pruning or topping was not a separately coded exclusion criterion. The resulting sample describes eligible trees at these sites rather than all administrative districts or age classes in Shanghai.

Tree, site, reproductive-trait and paired-temperature records were joined by tree and site identifiers. Fruit-ball production was represented by the integer tree counts in the original count field (n = 200). Adjusted analytical count fields were not substituted for direct counts because counts are discrete quantities. Only the 60 observation-block entries in the phenological pilot were summarized; expanded template entries and proposed airborne-monitoring fields were excluded from the study analyses.

### 2.2. Site Geolocation and Public-Source Urbanization Indicators

**Figure 1:**
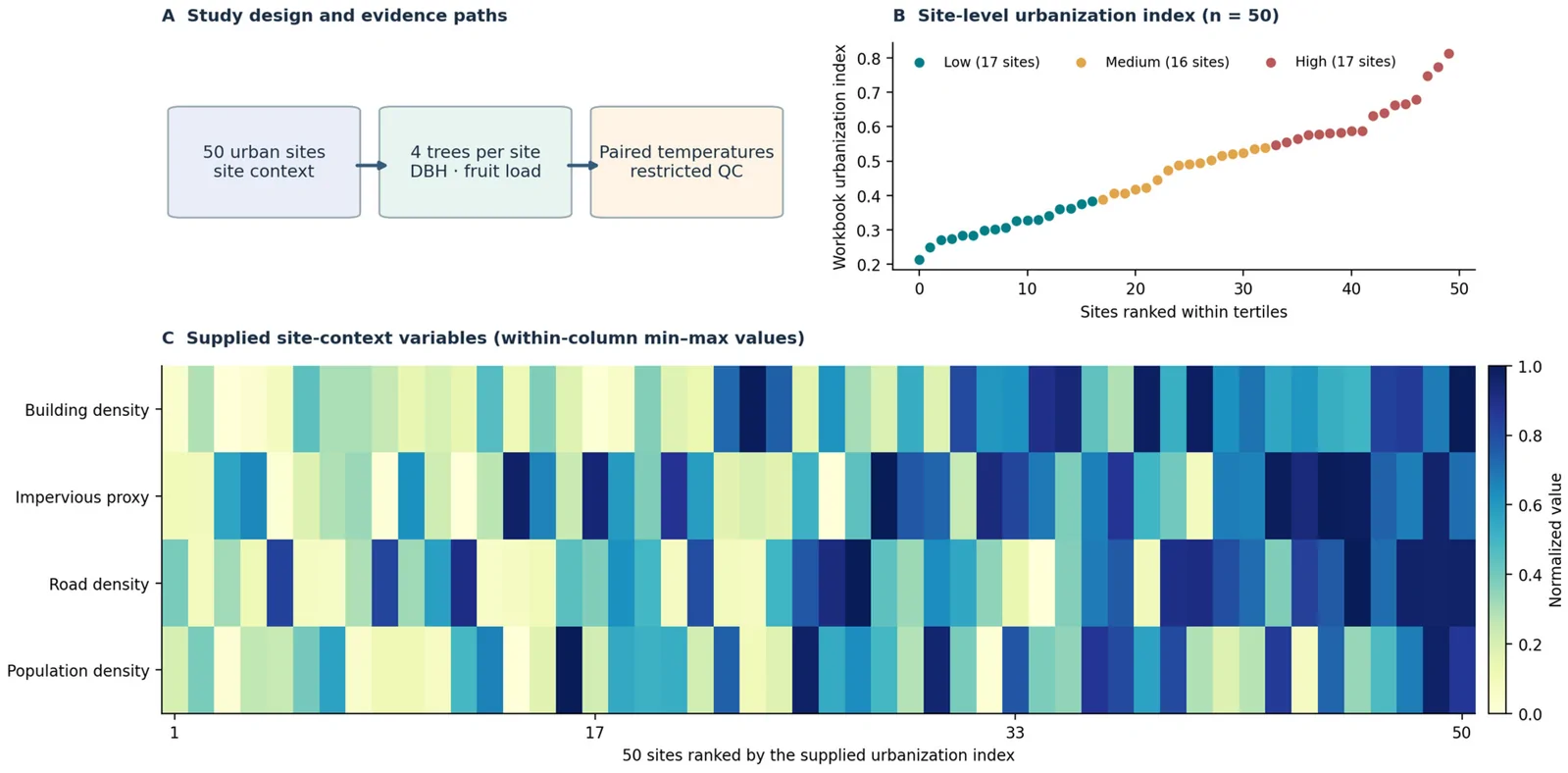
Study design and built-environment context. (**A**) Site and tree observations and the paired-temperature comparison. (**B**) Ranked site urbanization-index values. (**C**) Standardized site-context variables contributing to the descriptive gradient.

Site-level descriptors were obtained from the environmental inventory: building density, a built/impervious-surface proxy, road density and population density. The recorded composite urbanization index was used to form three site tertiles (17, 16 and 17 sites, corresponding to 68, 64 and 68 trees). All analyses use this tertile assignment (Figure 1). The index is a site-context descriptor, not a direct measurement of fruit-hair generation or human exposure.

**Figure 2:**
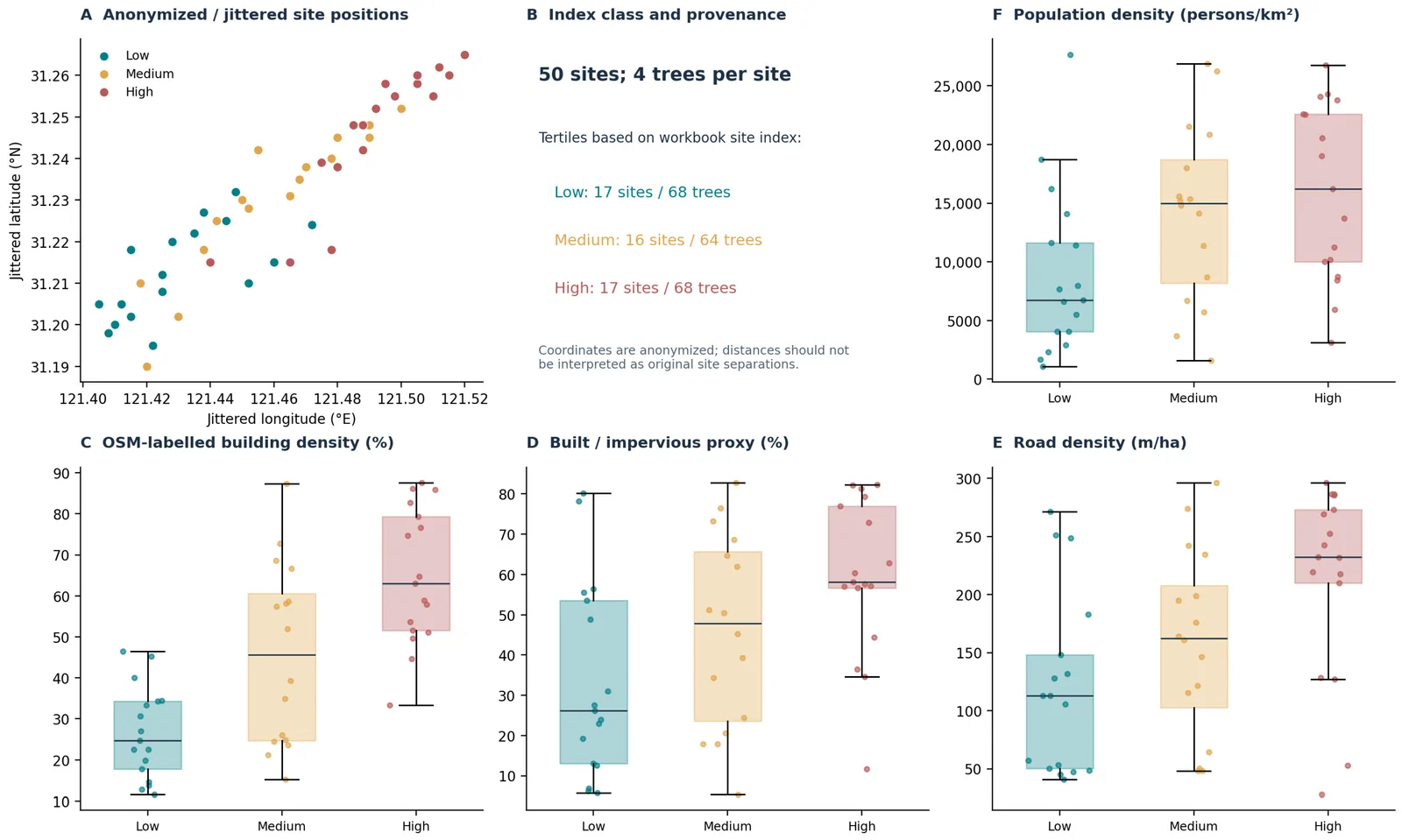
Site distribution and environmental context. (**A**) Anonymized locations of the 50 sites. (**B**) Site and tree counts by tertile. (**C**–**F**) Building density, built/impervious-surface proxy, road density and population density within each site class.

Figure 2 locates the 50 sites using anonymized coordinates and displays their environmental distributions. The coordinates are intended to illustrate the sample footprint, rather than to support claims of a particular minimum separation or comprehensive coverage of Shanghai districts. Site nesting was addressed in regression through site-clustered inference or site-level random intercepts. The two site-context summaries use overlapping geographic source variables and are not treated as independent validation datasets.

For descriptive comparison, we also calculated an equal-weight mean of the four min–max-scaled environmental variables. This reconstruction was highly correlated with the recorded index (r = 0.954), with a mean absolute difference of 0.047 and a maximum difference of 0.115. Consequently, the recorded composite, rather than the equal-weight reconstruction, was used consistently in the primary models. No inference about the independent causal effects of its individual components was made.

### 2.3. Tree Architecture, Maintenance Context and Field Instruments

Diameter at breast height (DBH; cm) was recorded at 1.3 m and ranged from 10 to 60 cm. Crown dimensions and tree height were available as contextual inventory fields, but DBH was selected a priori as the structural predictor for fruit-ball counts. The models therefore do not treat strongly related dimensions of the same tree as separate measures of reproductive development.

The site-level inventory also included pruning-event frequency (events yr^−1^), irrigation frequency and environmental descriptors. The pruning variable measures reported event frequency, not pruning season, removal intensity or the proportion of fruit-bearing branches removed; it was analysed as a site-management covariate rather than a treatment. Targeted fruit-bearing-branch pruning is documented in Shanghai management practice [16,17,18], independently of the associations examined here.

### 2.4. Fruit-Ball Load and Fruit-Hair Trait Measurements

Fruit-ball load was defined as the number of visible fruit balls recorded for each tree (n = 200). The original survey protocol described observations from four directions, using binocular assistance when necessary. Fruit-ball diameter and fruit mass were retained as descriptive variables. The reproductive count represents the tree as a whole and is distinct from microscopy-based subsampling of achene-associated hairs; microscopy replication was not used as an independent sample-size multiplier.

The laboratory trait table contains fibre length, diameter, density and mass descriptors. The analysis reports fibre length in millimetres and a mass entry in milligrams per laboratory-recorded unit; the latter is not assumed to equal either one achene or one entire fruit ball. Consequently, whole-tree released fibre mass cannot be inferred from this trait alone. Fruit hair, achene fibre and corresponding trichome measurements refer to achene-associated material in this study, not to leaf trichomes or pollen.

### 2.5. Cooling-Service, Shade and Seasonal Measurements

The thermal variable was defined as ΔT = T_open − T_under (°C) from paired readings in the service inventory. Positive ΔT indicates a lower recorded temperature at the under-canopy point at the observation occasion. No daylong, seasonal or thermal-comfort effect is derived from a single paired contrast. Because the retained data do not specify a common duration and observation-time window for all trees, the analysis is explicitly limited to paired field temperature contrasts.

The source field is recorded as “tree-shade coverage (%)”. We use the neutral label “shade-cover estimate” because the field does not specify a unique operational definition linking it to either ground-level shadow coverage or hemispherical canopy obstruction. The analysis treats this percentage as a recorded covariate, without extrapolating it to solar-radiation interception. Site soil moisture was included as a contextual predictor in temperature sensitivity models.

### 2.6. Temporal/Phenological Pilot and Airborne Fruit-Hair Monitoring Boundary

The phenological pilot comprises 10 tree identifiers observed in six named windows in 2025 (60 observation-block rows). These are treated as a descriptive sequence, not two independent growing years. For each tree, ΔT is identical across the six recorded windows, so the pilot contributes descriptive fruit-load, maturity and canopy information, but no independent estimate of seasonal change in temperature contrast.

This study did not include measured airborne fruit-hair concentrations, personal exposure, symptoms, complaint frequency or cleaning costs as response variables. These outcomes are distinguished from source-side traits throughout the manuscript.

### 2.7. Derived Indicators and Quality-Control Rules

The inventory includes calculated fruit-hair mass and a secondary contextual index. Both depend on component traits and analytical assumptions, so neither was used as a direct measure of airborne exposure or as an independent outcome in models containing its defining components. Where displayed, calculated hair mass is labelled as a derived descriptor. The former expression “relative exposure index” is not used as an exposure measure.

The 200 raw paired temperature differences had a mean of 5.49 ± 3.73 °C (range −3.1 to 14.6 °C); 12 were negative and 45 exceeded 8 °C. In addition to the full series, we examined a 0–8 °C restricted subset (143/200, 71.5%) and a 0–8 °C winsorized series (n = 200). The restriction was an analytical sensitivity definition rather than an instrument-error or biological threshold. All three representations are reported to show the consequences of restricting 28.5% of the original records.

### 2.8. Statistical Analysis

The tree was the observation unit, with 50 sites as clustering units. Fruit-ball count was fitted using negative-binomial (NB2) regression and site-clustered robust standard errors. DBH, site-level pruning frequency and the recorded urbanization index were each standardized by one sample standard deviation. Associations are expressed as incidence-rate ratios with 95% confidence intervals. Pairwise Pearson and Spearman correlations describe associations; direct count models exclude calculated fruit-hair mass as a response.

For temperature differences, we fitted ordinary least-squares models with site-clustered robust standard errors, including shade-cover estimate, urbanization index and soil moisture as predictors in the raw (n = 200), restricted (n = 143) and winsorized (n = 200) datasets. A site-random-intercept mixed model provided an additional analysis of the restricted subset. Coefficients refer to temperature degrees Celsius per one-standard-deviation increase in a covariate. The models estimate cross-sectional associations, not causal cooling effects.

The descriptive two-axis display combines fruit-ball count with temperature difference for the 143 records within 0–8 °C. Sample medians of 215 fruit balls and 4.8 °C define the quadrants. These sample-dependent categories are not operational pruning, retention or health-risk thresholds. No inferential seasonal test was applied to the 10-tree pilot.

## 3. Results

### 3.1. Urbanization-Context Indicators and Provenance Checks

The recorded urbanization-index tertiles comprised 17 low-, 16 medium- and 17 high-urbanization sites (68, 64 and 68 trees, respectively; Table 1 and Figure 1). Mean index values were approximately 0.31, 0.47 and 0.63, respectively. Building and road density increased across the tertiles, with appreciable variation within groups. This index is used solely to describe the environmental context of the sampled streets.

The site and tree allocations were consistent under the selected index-tertile definition (Table 2; Figure 2). The corresponding counts in the 0–8 °C thermal subset were 46, 50 and 47 trees for low-, medium- and high-urbanization sites, respectively. Figure 2 shows the distribution of site-context variables alongside an anonymized representation of the site locations; no inferential spatial-separation rule was applied.

### 3.2. Fruit-Ball Counts, Hair Traits and Paired Temperature Descriptors Across Sites

**Figure 3:**
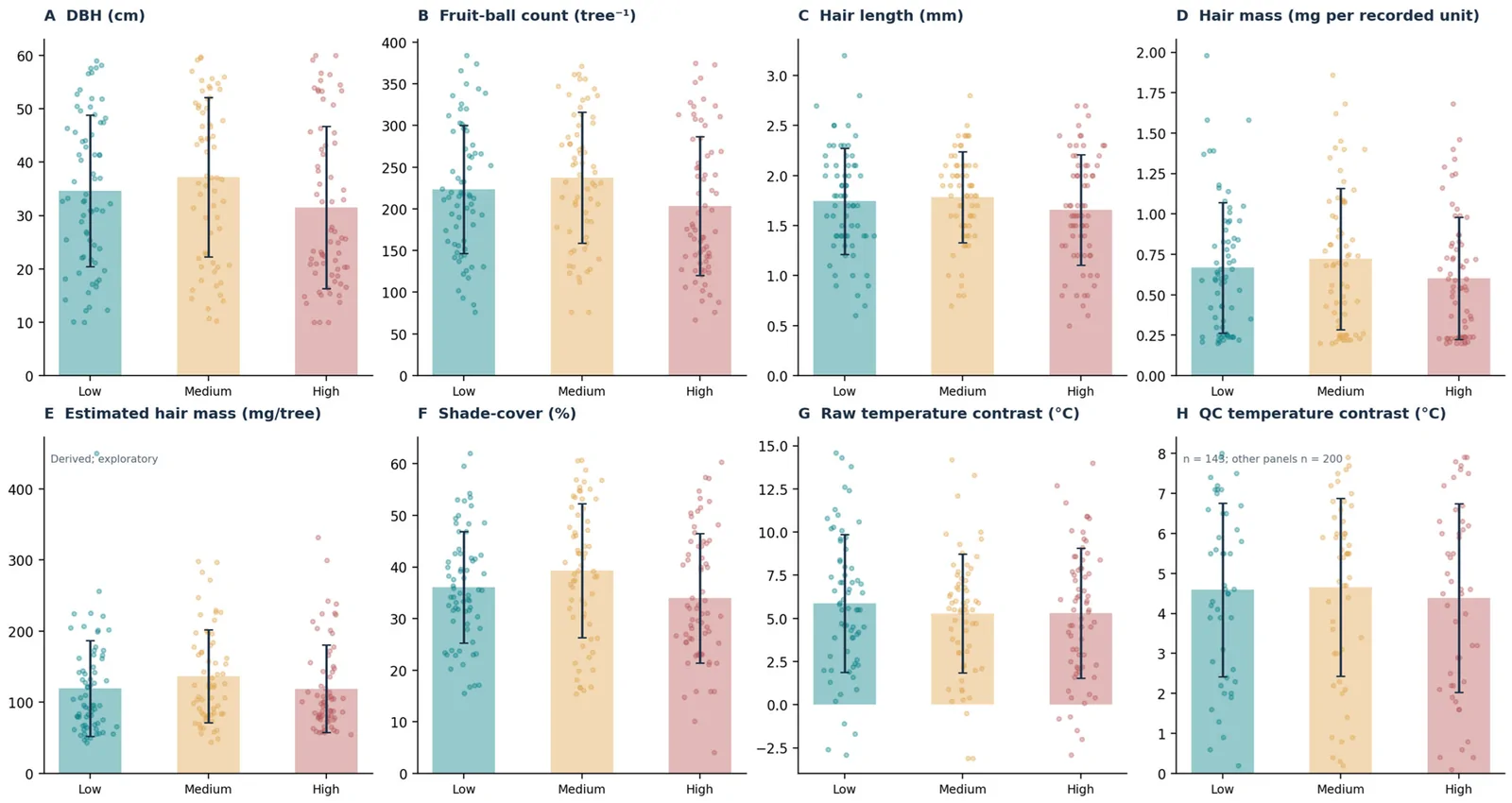
Tree diameter, directly recorded fruit-ball counts, fibre traits and paired temperature differences across the site-index tertiles. The calculated hair-mass field in Panel (**E**) is descriptive. Panel (**G**) uses 200 raw temperature differences; Panel (**H**) uses 143 temperature differences between 0 and 8 °C. Bars show means ± SD; points represent trees.

Fruit-ball counts differed in descriptive means across the three site strata (Table 3; Figure 3). Mean counts were 223.3 ± 77.0, 237.5 ± 78.8 and 203.6 ± 83.5 per tree for low-, medium- and high-index tertiles, respectively. Fibre length and laboratory-recorded fibre mass did not increase monotonically with the index. The recorded temperature difference was evaluated for all 200 trees and separately for the 143-record restriction; the restricted group sizes were 46, 50 and 47.

### 3.3. Original Fruit-Ball Counts Were Closely Correlated with DBH

**Figure 4:**
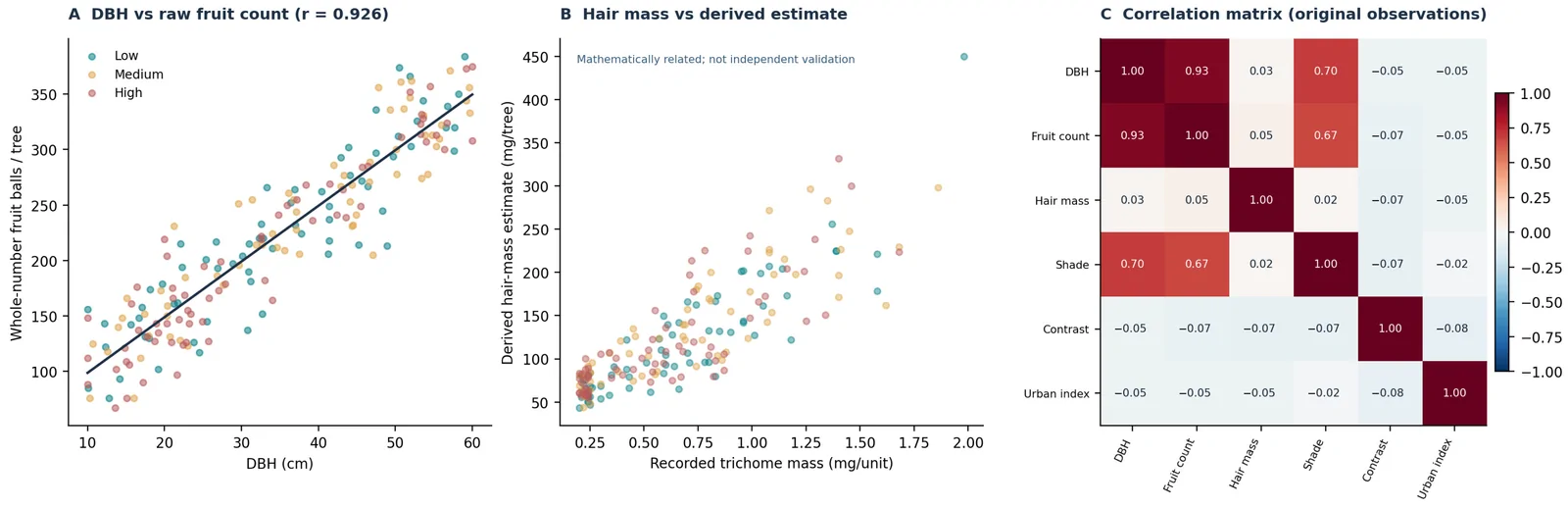
Tree-structure and reproductive-trait correlations. (**A**) DBH versus directly recorded fruit-ball count. (**B**) Fibre mass per recorded laboratory unit versus calculated hair mass; this association is partly definitional. (**C**) Correlation matrix of recorded traits and environmental descriptors.

DBH was strongly associated with fruit-ball count (Pearson r = 0.926; Spearman r = 0.923; n = 200; Table 4; Figure 4A). This association describes the recorded inventory, but does not establish a threshold DBH for reproductive maturity or isolate crown size, age and branch history as potential mechanisms. The sampled range was 10–60 cm DBH.

The urbanization index had only a weak bivariate association with fruit-ball count (Pearson r = −0.054), as did site-level pruning frequency (r = 0.037). Laboratory-recorded fibre mass was correlated with calculated hair mass (r = 0.851), but this association is partly definitional. Fruit-ball count and raw paired temperature difference were weakly related (r = −0.068). Thus, direct size–count evidence is interpreted separately from correlations involving a calculated indicator.

### 3.4. Count and Thermal Models: Primary Estimates and Sensitivity Checks

Negative-binomial regression showed that DBH was associated with fruit-ball count (IRR 1.419 per one SD, 95% CI 1.385–1.454; *p* < 0.001; Table 5). The corresponding coefficients for the site urbanization index (IRR 0.989, *p* = 0.228) and pruning-event frequency (IRR 1.006, *p* = 0.576) were not statistically significant. The count-model estimates, rather than coefficients from a Gaussian response model, provide the main fruit-ball inference. All associations remain observational.

**Figure 5:**
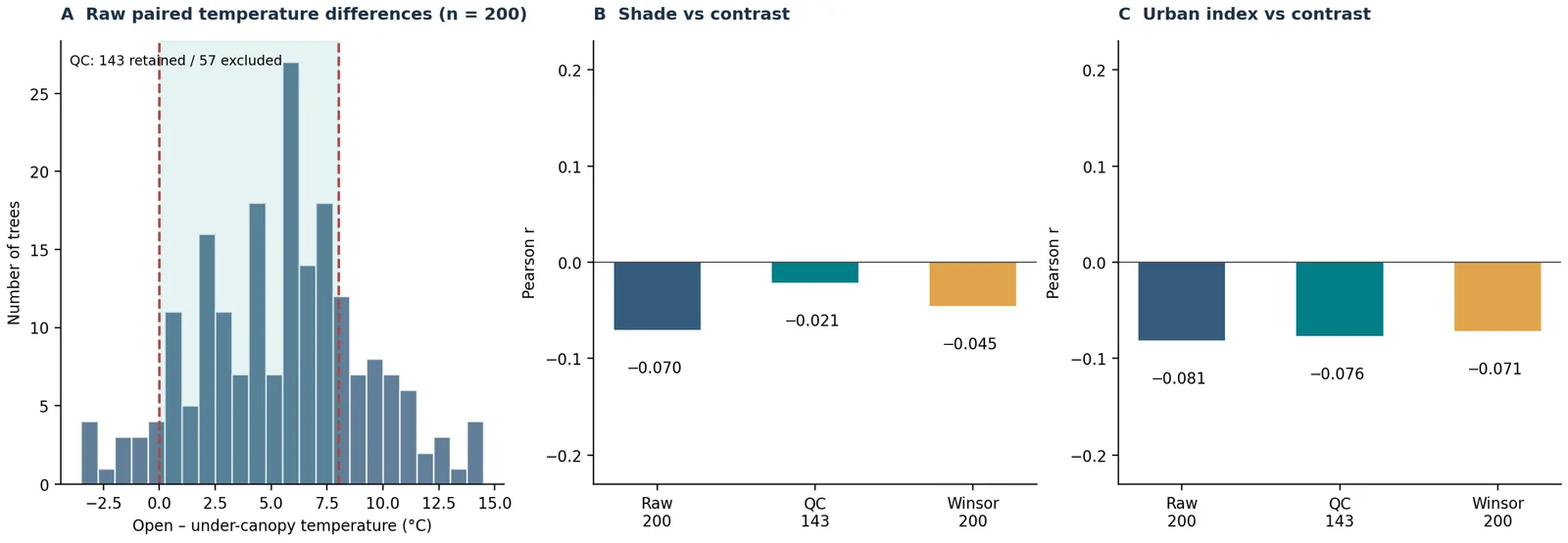
Distributional and model-sensitivity assessment of paired temperature differences. (**A**) Raw temperature-difference distribution with the 0–8 °C sensitivity interval. (**B**,**C**) Pearson associations with shade-cover estimate and urbanization index under the raw, restricted and winsorized treatments.

The shade-cover estimate was not significantly correlated with ΔT under any of the three temperature-data definitions (Table 6; Figure 5): Pearson r = −0.070 for raw data (n = 200), −0.021 for the 0–8 °C subset (n = 143) and −0.045 for winsorized data (n = 200). Site-cluster-robust multivariable shade coefficients were also not significant in all three treatments. In the restricted mixed model, the shade coefficient was −0.036 °C per SD (*p* = 0.848).

### 3.5. The Recorded Shade-Cover Estimate Did Not Predict a Robust Thermal Contrast

The full inventory yielded a mean paired temperature difference of 5.49 ± 3.73 °C (n = 200); the 0–8 °C subset averaged 4.55 ± 2.24 °C (n = 143). Removal of 57 records under the range restriction did not produce a significant positive shade-cover relationship. These values represent observed paired-location contrasts, not integrated cooling over a day, season or pedestrian experience.

### 3.6. Exploratory Joint Screen Using Direct Fruit-Ball Load

**Figure 6:**
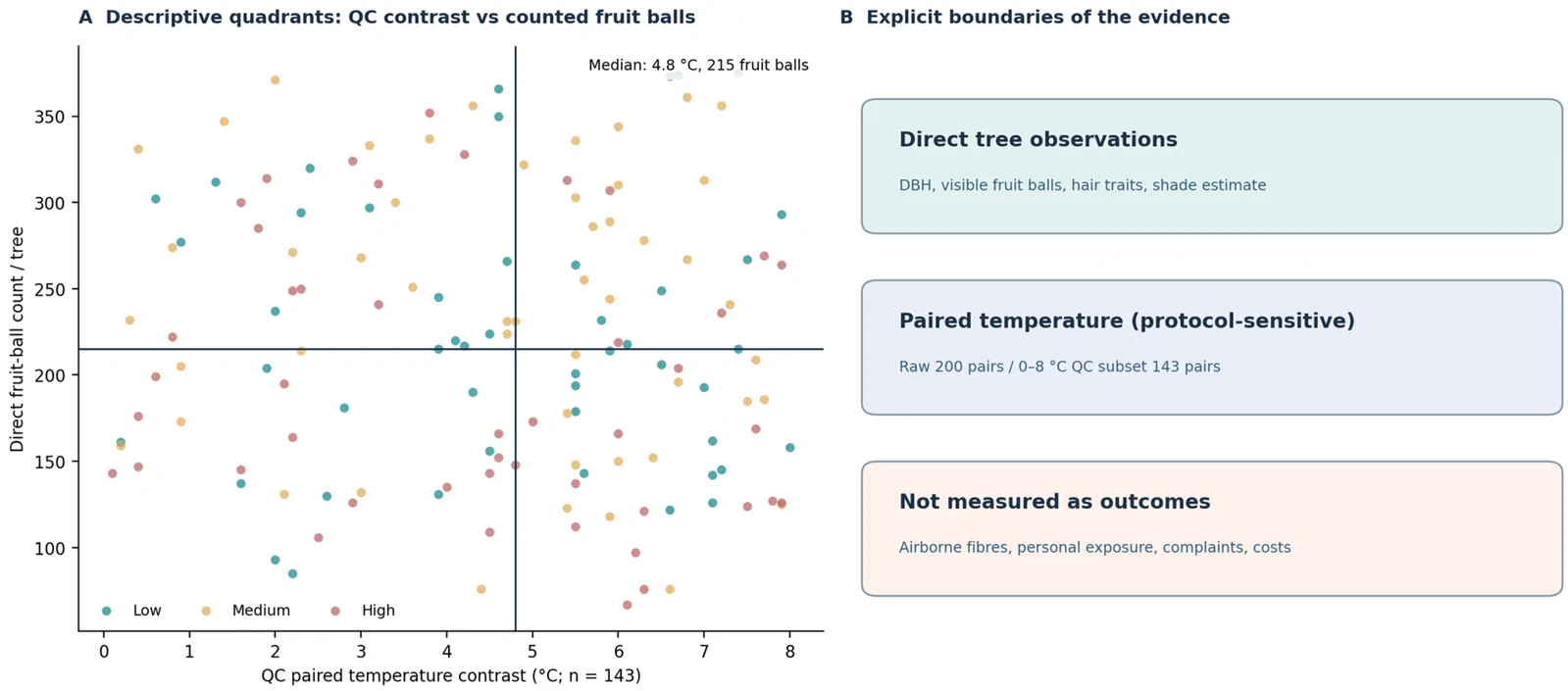
Exploratory joint distribution of paired temperature contrast and fruit-ball count. (**A**) Tree-level observations in the 0–8 °C subset, separated by sample medians and coloured by site-index tertile. (**B**) Direct structural observations, derived descriptors and outcomes outside the measurements (airborne fibres, human exposure, complaints and maintenance cost).

Within the 143 temperature records between 0 and 8 °C, the median was 4.8 °C and the median fruit-ball count was 215 per tree. Four descriptive categories contained 32, 40, 40 and 31 trees (Table 7; Figure 6). These groupings summarize co-occurring temperature differences and reproductive loads within the study subset; they are not validated evidence of a cooling–fruit-hair trade-off or decision thresholds for tree removal or pruning.

### 3.7. Limited Multi-Window Observations from the 10-Tree Pilot

The phenological pilot summarized 10 trees across six observation windows (Table 8). Recorded fruit-ball counts, maturity scores and canopy descriptors varied among the windows, whereas the open-minus-under-canopy temperature difference was identical for each tree across the six windows, with a mean of 5.10 °C per window. Accordingly, the pilot describes changes in recorded phenological traits, but provides no independent seasonal contrast in ΔT.

## 4. Discussion

### 4.1. Platanus Tree Structure and the Source of Seasonal Fruit Material

**Figure 7:**
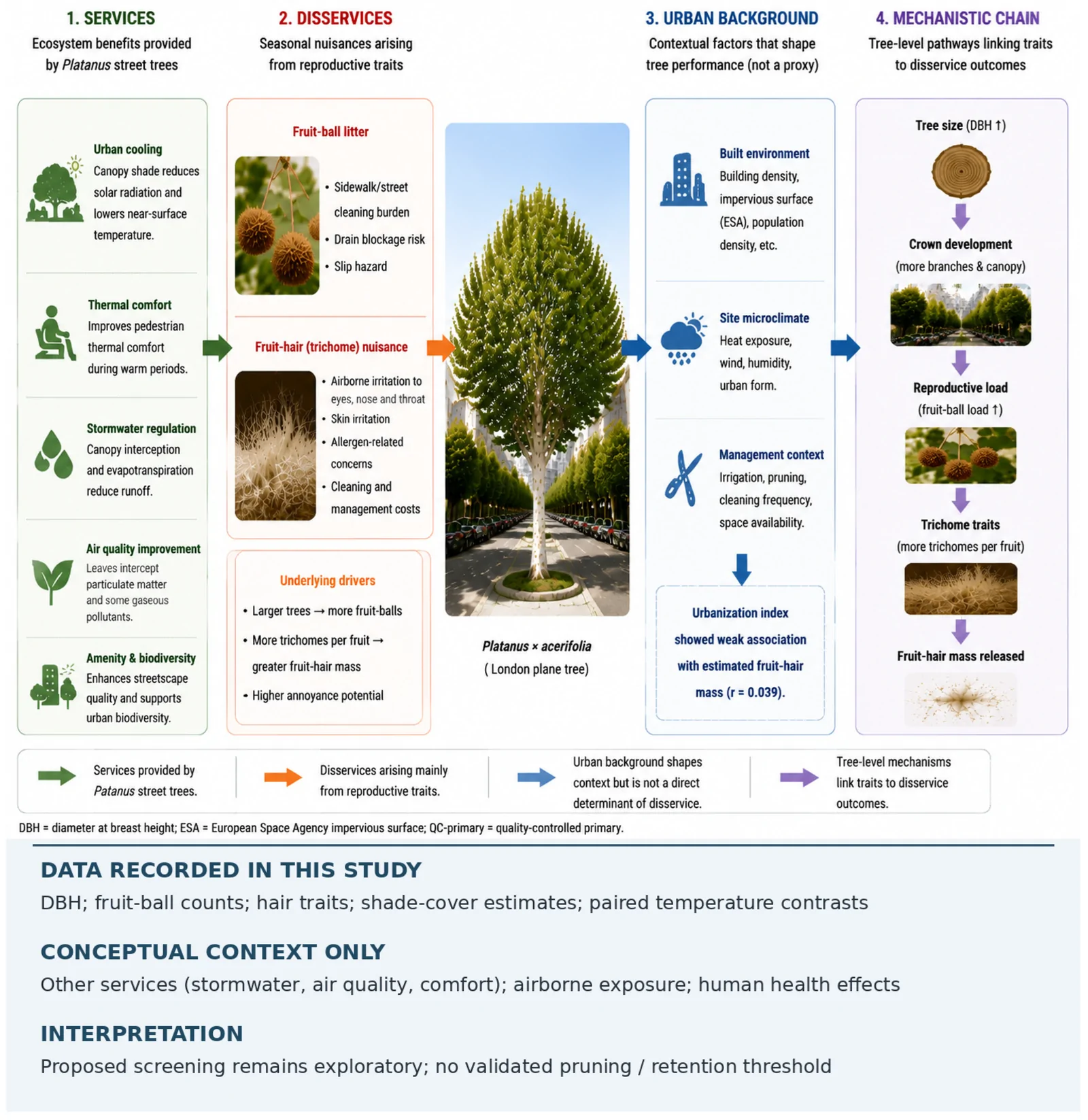
Conceptual relationships between *Platanus* ecosystem services and seasonal reproductive material. Measured variables in this study comprise DBH, recorded fruit-ball counts, laboratory hair traits, shade-cover estimates and paired temperature differences. Stormwater regulation, air-quality improvement, thermal comfort, airborne exposure and health endpoints are wider contextual considerations, not outcomes tested here.

The clearest relationship in the inventory was between DBH and fruit-ball count, whereas the site-level urbanization index was weakly related to reproductive load. Larger trees may have greater potential fruit-bearing branch area, offering a biologically plausible explanation for this pattern. Nevertheless, tree diameter is correlated with other unmeasured components of reproductive development and management history, so the observed coefficient is not itself evidence for a threshold of reproductive maturity. The conceptual illustration (Figure 7) separates observations in this study from broader services associated with urban trees.

### 4.2. Reproductive Traits and the Limits of Derived Source-Burden Metrics

Tree-level fruit-ball counting is more directly connected to reproductive load than a broad site urbanization index. Laboratory traits add useful morphological description, but the anatomical denominator of the mass field is not established within the analysis dataset; accordingly, we report its recorded units without extrapolating to released mass per tree. Calculated hair-mass descriptors cannot be independently explained by mathematical components used to construct them. No airborne fibre concentration, personal exposure or complaint outcome was measured, so this study does not infer these endpoints from source-side predictors.

### 4.3. Urbanization, Targeted Pruning and Spatial Interpretation

The urbanization index characterized the sampled environments, but did not independently predict direct fruit-ball counts after accounting for DBH and pruning-event frequency (NB2 *p* = 0.228). Site-level pruning frequency was also not significant (*p* = 0.576). This result concerns the frequency variable in the inventory and does not test the effects of removing fruit-bearing branches, an established Shanghai control practice [16,17,18]. The anonymized spatial representation provides geographic context without establishing district-wide representativeness or a minimum site distance.

### 4.4. Paired Temperature Observations Are Not Calibrated Whole-Day Cooling

Across raw, restricted and winsorized records, the measured shade-cover percentage did not exhibit a significant positive association with the paired temperature difference. The sensitivity analysis is important because a 0–8 °C restriction excludes 57 of 200 observations and changes the empirical distribution. Short-duration paired measurements are influenced by observation time, solar conditions, wind and reference-point selection. The results therefore describe the recorded comparisons rather than daylong tree cooling, and they neither confirm nor contradict the established physical cooling mechanisms documented in other settings [3,4,5,6,11,12,13,25].

### 4.5. Fruit-Hair Fibres, Pollen and Directly Measured Exposure

Studies on *Platanus* pollen, Pla a 1 and airborne allergenicity [19,20,21,22,23] concern exposures that differ from the achene-associated hair source examined here. Airborne *Platanus* bioaerosol components have been reported elsewhere [24], but fruit-ball count and microscopic fibre traits do not directly measure concentrations inhaled by pedestrians. This inventory also contains no resident complaints, health outcomes or documented cleaning costs. Inference is therefore restricted to reproductive production and paired temperature observations.

### 4.6. Scope of Inference and Future Field Measurements

This study concerns apparently healthy street trees at 50 selected Shanghai sites and DBH values of at least 10 cm. Results should not be generalized to trees below this threshold, visibly diseased or damaged trees or extensively topped trees. The temperature metric is a recorded paired contrast without a common observation period, and the shade-cover percentage is not a calibrated solar-shading measure. These features delimit the interpretation of the current analysis. Future work can combine repeated time-standardized thermal monitoring, directly sampled airborne fruit hairs, detailed pruning histories and resident/maintenance records to develop and externally assess management thresholds.

## 5. Conclusions

Across 200 *Platanus* × *acerifolia* street trees at 50 Shanghai sites, DBH was positively associated with counted fruit balls (NB2 IRR 1.419 per one SD, 95% CI 1.385–1.454), whereas site urbanization and pruning-event frequency were not significant predictors in the fitted model. The calculated fruit-hair mass field was distinguished from observed airborne concentration, and this study does not infer human exposure from fruit-ball counts.

The 200 raw paired temperature differences averaged 5.49 °C, while the 143 records within 0–8 °C averaged 4.55 °C. Shade-cover estimates were not consistently associated with these differences. Sample-median temperature–fruit-count categories can describe combinations of recorded tree attributes, but do not establish a trade-off or an operational pruning priority. Application beyond the observed healthy-tree and diameter range will require additional sampling; management thresholds should be developed from repeated thermal observations and direct airborne, resident and maintenance outcomes.

## Figures and Tables

**Table 1:** Site-level environmental descriptors across urbanization-index tertiles (mean ± SD; 17, 16 and 17 sites).

Indicator	Low	Medium	High
Sites (n)	17	16	17
Building density (%)	26.56 ± 11.16	45.68 ± 22.17	64.78 ± 16.56
Built/impervious proxy (%)	33.41 ± 24.25	45.91 ± 24.08	59.51 ± 19.50
Road density (m ha^−1^)	119.72 ± 78.02	158.46 ± 80.31	214.06 ± 81.92
Population density (persons km^−2^)	8837.26 ± 7102.95	14,148.77 ± 7580.66	15,948.42 ± 7506.28
Supplied urbanization index	0.31 ± 0.05	0.47 ± 0.05	0.63 ± 0.08

Note. The three classes are tertiles of the recorded site urbanization index. They describe the built-environment and demographic setting of the inventory, rather than directly measured fibre production or exposure.

**Table 2:** Sample allocation, temperature-subset retention and spatial presentation by urbanization tertile.

Descriptor	Low	Medium	High
Study sites	17	16	17
Inventoried trees	68	64	68
Share of inventoried trees	34.0%	32.0%	34.0%
0–8 °C subset, trees	46	50	47
Subset retention (%)	67.6%	78.1%	69.1%
Location display	Anonymized	Anonymized	Anonymized

Note. Tertile membership is defined by the recorded site-level urbanization index. The mapped point positions are anonymized and are provided for geographic orientation only.

**Table 3:** Tree structure, reproductive traits and paired temperature observations across site strata (mean ± SD).

Indicator	Low	Medium	High
n trees	68	64	68
Index value	0.31 ± 0.05	0.47 ± 0.05	0.63 ± 0.08
DBH (cm)	34.64 ± 14.18	37.19 ± 14.92	31.54 ± 15.20
Fruit-ball count (tree^−1^)	223.34 ± 76.96	237.55 ± 78.80	203.59 ± 83.47
Fibre length (mm)	1.74 ± 0.53	1.78 ± 0.45	1.66 ± 0.55
Fibre mass (mg per laboratory-recorded unit)	0.67 ± 0.40	0.72 ± 0.44	0.60 ± 0.38
Calculated fruit-hair mass field (mg tree^−1^)	119.66 ± 67.49	136.65 ± 65.41	119.03 ± 61.56
Shade-cover estimate (%)	36.07 ± 10.77	39.29 ± 13.01	33.98 ± 12.55
Raw paired temperature ΔT (°C)	5.87 ± 3.98	5.28 ± 3.43	5.30 ± 3.76
Restricted paired ΔT (°C)	4.59 ± 2.17	4.66 ± 2.22	4.39 ± 2.36

Note. Tree-level group sizes are 68, 64 and 68 except for the 0–8 °C paired-temperature subset (46, 50 and 47). Fibre mass is stated per laboratory-recorded unit, without assigning it to a whole fruit ball or single achene. The calculated hair-mass field is descriptive and is not measured airborne material.

**Table 4:** Pearson and Spearman associations among tree attributes, environmental context and thermal records.

Variable 1	Variable 2	n	Pearson r	Spearman r	Interpretation
DBH	Fruit-ball count	200	0.926	0.923	Original count
Urbanization index	Fruit-ball count	200	−0.054	−0.080	Context vs. original count
Pruning frequency	Fruit-ball count	200	0.037	0.036	Site management descriptor
Shade-cover estimate	Paired ΔT, raw	200	−0.070	−0.034	Raw 200 pairs
Urbanization index	Paired ΔT, raw	200	−0.081	−0.075	Raw 200 pairs
Fruit-ball count	Paired ΔT, raw	200	−0.068	−0.050	Original count vs. ΔT
Fibre mass per recorded unit	Calculated fruit-hair mass	200	0.851	0.856	Calculated indicator; partly definitional
Fruit-ball count	Calculated fruit-hair mass	200	0.481	0.480	Calculated indicator; exploratory
Calculated hair mass	Calculated fruit-hair mass	200	−0.082	−0.071	Calculated indicator; descriptive
Shade-cover estimate	Paired ΔT, restricted	143	−0.021	−0.016	0–8 °C sensitivity subset
Urbanization index	Paired ΔT, restricted	143	−0.076	−0.046	0–8 °C sensitivity subset

Note. n = 200 for full-inventory pairs and n = 143 for the 0–8 °C temperature subset. Correlations involving the calculated hair-mass field are descriptive only because its definition includes component traits.

**Table 5:** Primary NB2 fruit-ball model and restricted-sample mixed-effects thermal model.

Model and Response	Predictor	Effect (95% CI)	*p* Value	n/Sites	Inference
NB2: whole fruit balls	DBH, per SD	IRR 1.419 (1.385–1.454)	<0.001	200/50	Cluster-robust
NB2: whole fruit balls	Urbanization index, per SD	IRR 0.989 (0.971–1.007)	0.228	200/50	Cluster-robust
NB2: whole fruit balls	Pruning frequency, per SD	IRR 1.006 (0.985–1.028)	0.576	200/50	Cluster-robust
Mixed: ΔT restricted	Shade cover, per SD	−0.036 (−0.408 to +0.336) °C	0.848	143/50	Site random intercept
Mixed: ΔT restricted	Urbanization index, per SD	−0.166 (−0.542 to +0.211) °C	0.388	143/50	Site random intercept
Mixed: ΔT restricted	Soil moisture, per SD	−0.038 (−0.414 to +0.339) °C	0.844	143/50	Site random intercept

Note. NB2 regression uses n = 200 integer counts and 50 site clusters; results are IRRs for one-SD predictor changes, with site-clustered standard errors. The restricted temperature mixed model includes n = 143 pairs across 50 sites and reports slopes in °C per predictor SD. Confidence intervals are 95%.

**Table 6:** Sensitivity of shade-cover and urbanization associations to treatment of paired temperature differences.

Thermal Dataset	n	Shade Pearson r	Urbanization Pearson r	Shade Adjusted Slope °C/SD (*p*)	Urbanization Slope °C/SD (*p*)
Raw (unfiltered)	200	−0.070	−0.081	−0.268 (0.300)	−0.299 (0.285)
Restricted: 0–8 °C	143	−0.021	−0.076	−0.047 (0.817)	−0.170 (0.402)
Winsorized: 0–8 °C	200	−0.045	−0.071	−0.126 (0.540)	−0.189 (0.326)

Note. Temperature-difference response and adjusted coefficients are in °C. Predictors were shade-cover estimate, urbanization index and soil moisture, with site-clustered robust OLS inference. The 0–8 °C range was evaluated as a sensitivity subset, not as an error-detection threshold.

**Table 7:** Sample-median classification of temperature contrast and direct fruit-ball count (n = 143).

Quadrant (0–8 °C Subset)	Total	Low	Medium	High	Definition
High ΔT/high fruit count	32	8	16	8	At/above versus below corresponding sample medians
High ΔT/low fruit count	40	13	13	14	At/above versus below corresponding sample medians
Low ΔT/high fruit count	40	15	14	11	At/above versus below corresponding sample medians
Low ΔT/low fruit count	31	10	7	14	At/above versus below corresponding sample medians

Note. Horizontal split: 4.8 °C paired temperature difference; vertical split: 215 counted fruit balls per tree. The column totals are 46, 50 and 47 for low-, medium- and high-urbanization sites, respectively. Medians define relative sample classes rather than standardized management thresholds.

**Table 8:** Descriptive phenological pilot records for 10 trees across six observation windows (mean ± SD).

Window Group	Window Description	Window Number	n Trees	Fruit Balls	Maturity Score	LAI	Shade (%)	Under T (°C)	Open T (°C)	ΔT (°C)
Window block 1	Pre-release/early season	1	10	240.00 ± 88.25	3.20 ± 0.42	1.02 ± 0.41	38.97 ± 12.37	26.21 ± 1.71	31.31 ± 1.10	5.10 ± 1.14
Window block 1	Peak release/mid season	2	10	210.50 ± 83.85	5.00 ± 0.00	1.10 ± 0.42	41.17 ± 12.48	27.01 ± 1.71	32.11 ± 1.10	5.10 ± 1.14
Window block 1	Post-peak/late season	3	10	170.80 ± 77.82	4.00 ± 0.00	1.19 ± 0.45	43.51 ± 12.78	26.71 ± 1.71	31.81 ± 1.10	5.10 ± 1.14
Window block 2	Vegetative/early fruit	4	10	229.80 ± 86.11	2.30 ± 0.48	1.06 ± 0.41	39.87 ± 12.47	29.01 ± 1.71	34.11 ± 1.10	5.10 ± 1.14
Window block 2	Mid-season	5	10	245.80 ± 87.82	3.00 ± 0.00	1.11 ± 0.43	41.28 ± 12.58	29.81 ± 1.71	34.91 ± 1.10	5.10 ± 1.14
Window block 2	Late season	6	10	204.20 ± 81.04	2.00 ± 0.00	0.99 ± 0.39	38.00 ± 12.06	29.51 ± 1.71	34.61 ± 1.10	5.10 ± 1.14

Note. Each window contains 10 tree-level entries. Window blocks belong to 2025 and are not two independent growing years. Because each tree has the same recorded ΔT across windows, no seasonal ΔT test was conducted.

## Data Availability

The data supporting the findings of this study are part of an ongoing research project that has not yet been completed. Consequently, the data are not deposited in a public repository at this stage. Data supporting the results reported in this article are available from the corresponding author upon reasonable request.
